# Cost-Effectiveness Analysis of the Bivalent and Quadrivalent Human Papillomavirus Vaccines from a Societal Perspective in Colombia

**DOI:** 10.1371/journal.pone.0080639

**Published:** 2013-11-18

**Authors:** Johanna Aponte-González, Luisa Fajardo-Bernal, Jorge Diaz, Javier Eslava-Schmalbach, Oscar Gamboa, Joel W. Hay

**Affiliations:** 1 Clinical Research Institute, Clinical Epidemiology Department, School of Medicine, Universidad Nacional de Colombia, Bogotá, Colombia; 2 Pharmacology Department, School of Pharmacy, Universidad Nacional de Colombia, Bogotá, Colombia; 3 Research Department (Subdirección Investigaciones), Instituto Nacional de Cancerología de Colombia, Bogotá, Colombia; 4 Titus Family Department of Clinical Pharmacy and Pharmaceutical Economics and Policy, School of Pharmacy, University of Southern California, Los Angeles, California, United States of America; Baylor College of Medicine, United States of America

## Abstract

**Objective:**

To compare costs and effectiveness of three strategies used against cervical cancer (CC) and genital warts: (i) Screening for CC; (ii) Bivalent Human Papillomavirus (HPV) 16/18 vaccine added to screening; (iii) Quadrivalent HPV 6/11/16/18 vaccine added to screening.

**Methods:**

A Markov model was designed in order to simulate the natural history of the disease from 12 years of age (vaccination) until death. Transition probabilities were selected or adjusted to match the HPV infection profile in Colombia. A systematic review was undertaken in order to derive efficacy values for the two vaccines as well as for the operational characteristics of the cytology test. The societal perspective was used. Effectiveness was measured in number of averted Disability Adjusted Life Years (DALYS).

**Results:**

At commercial prices reported for 2010 the two vaccines were shown to be non-cost-effective alternatives when compared with the existing screening strategy. Sensitivity analyses showed that results are affected by the cost of vaccines and their efficacy values, making it difficult to determine with certainty which of the two vaccines has the best cost-effectiveness profile. To be ‘cost-effective’ vaccines should cost between 141 and 147 USD (Unite States Dollars) per vaccinated girl at the most. But at lower prices such as those recommended by WHO or the price of other vaccines in Colombia, HPV vaccination could be considered very cost-effective.

**Conclusions:**

HPV vaccination could be a convenient alternative for the prevention of CC in Colombia. However, the price of the vaccine should be lower for this vaccination strategy to be cost-effective. It is also important to take into consideration the willingness to pay, budgetary impact, and program implications, in order to determine the relevance of a vaccination program in this country, as well as which vaccine should be selected for use in the program.

## Introduction

CC is a significant health issue in Colombia. According to the National Cancer Institute every year there are 5,500 new cases of CC in the country [1]. For 2002, 2,045 deaths due to malignant cervical tumors were reported, making CC a leading cause of death in women, above breast and gastric cancer [2].

The screening program implemented in 1991 has contributed to a decrease in mortality from this cause over the past 7 years. However, this decrease is less than originally expected.

Epidemiological studies conducted in the 1990's provided evidence on the role of the HPV in the etiology of CC[3,4]. This led to the introduction of two HPV vaccines in recent years: a bivalent vaccine that prevents infection by types 16 and 18, and a quadrivalent vaccine against HPV 06/11/16/18. 

According to the World Health Organization (WHO), it is estimated that developing countries, with a high incidence of CC and difficulty implementing screening programs, are the ones that may derive the greatest benefit from vaccination against the human papillomavirus [5]. For Colombia, local studies have detected quality problems in screening[6], and even though coverage is around 70%[7], technical and economic resources are not being correctly allocated to ensure quality of the testing or appropriate follow-up of abnormal results [8].

Hence, an in-depth analysis of the issue is necessary, in order to evaluate the convenience of vaccination with one of the two types of vaccines available. Such analysis must be based on the evidence available so far and consider the unique characteristics of the national context such as the prevalence of infections by the different HPV types, the coverage of the vaccination programs, and the cost of the vaccine in the domestic market, among others.

Consequently, the goal of this work is to compare the cost and the effectiveness of HPV vaccines in the Colombian context. 

## Materials and Methods

### Ethics Statement

This project was approved by the independent ethics committee of Universidad Nacional de Colombia – Medicine School. Since this project uses secondary data, no ethical concerns exist.

### Model Design

A Markov model was developed in order to represent the natural history of CC and genital warts, based on prior models of the disease accessed through a systematic search in Pubmed-Medline, Embase and Lilacs. The model and the validity of the assumptions were then reviewed by gynecologists, gynecological oncologists and experts in decision-making models. The specialized TreeAge Pro 2011® software was used to build the model.

**Figure 1:**
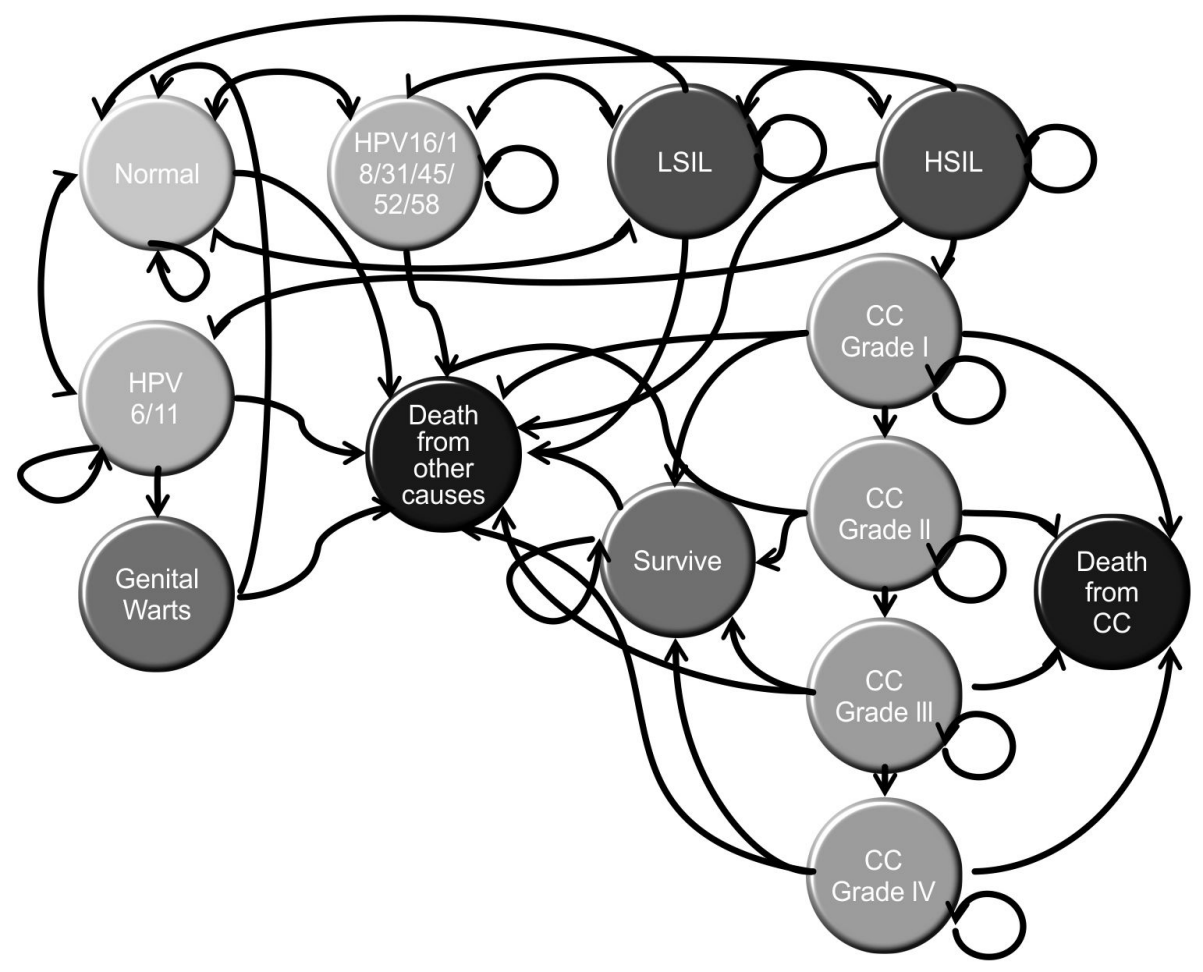
Simplified model of the disease. Markov model of disease is represented. Circles correspond to the states and arrows represent the allowed transitions. HPV: Human papillomavirus; LSIL: Low-grade Squamous Intraepithelial Lesion; HSIL: High-grade Squamous Intraepithelial Lesion; CC: Cervical Cancer. Source: Author.

The model was designed as described in Figure 1. It consists of 13 mutually exclusive health states that represent the different events throughout the natural history of the disease. It was refined using models found in the relevant literature [9-13]. In the analysis, the cohort would start at 12 years of age, the age at which the women would be vaccinated. This age was chosen in accordance with the WHO recommendation for starting vaccination [5]. Using yearly cycles, the women in the cohort would go from one state to another in accordance with the transition probabilities that govern the model. The model was set to run until the cohort was 76 years old, according to life expectancy for women in Colombia. 

The following assumptions were used for the sake of simplification:

- All cases of genital warts are diagnosed and treated within the same year, and treatment efficacy is 100%.
- Only healthy women may become infected with HPV 6/11 and, consequently, acquire genital warts.
- The screening approach for all women who have access to it is called 1-1-3 program, which means that after two negative cytology test results, they wait three years for testing. Otherwise, they should come back the following year for another cytology test [14].
- For cancer stages I and II, treatment will be given to those women who are identified by screening or who develop symptoms that prompt them to seek medical care. For cancer stages III and IV, considering that the probability of symptoms appearing is 0.6 and 0.9, the model was simplified by bypassing screening as a tool for diagnosing the disease.

This model will measure costs in US Dollars and effectiveness in averted DALYS (Disability-Adjusted Life Years), in accordance with the WHO recommendation[15]. DALYS are defined only for CC stages. For other diseases such as Genital Warts, Low-grade Squamous Intraepithelial Lesion (LSIL) or High-grade Squamous Intraepithelial Lesion (HSIL) DALYS have not been defined. 

### Model Parameters

Table 1 describes the costs as well as the transition probabilities and all other parameters of the model.

**Table 1:** Parameters of the model.

**1. TRANSITION PROBABILITIES**								
**Age**	**Normal-HPV HR**	**Normal-HPV LR**	**Normal -> VPH 6/11**	**VPH LR -> Normal**	**VPH LR-Normal**			**Reference**
16	0.10	0.0135	0.018	0.5518	**0.82**			
85	0.00045	0.055	0	0.135157	**0.82**			[16,17,61]
**HPV-> LSIL**								**Reference**
**Age**	**No Vaccine**	**Protected HR16/18**	**HPV LR**	**Protected HPV HR**	**Min**	**Max**		
15	0.072	0.0706	0.036	0.076	0.014	0.14		
85	0.072	0.0706	0.036	0.076	0.14	0.14		[11,13,61]
**HPV -Warts**			**Warts-Normal**					**Reference**
**Base Case**	**Max**	**Max**	**BaseCase**	**Max**	**Min**			
0.300	0.027	0.57	0.875	0.875	0.875			[9,61,62]
**HPV-HSIL**								**Reference**
**Age**	**No Vaccination**	**Protected HPV 16/18**	**Protected HPV HR**					
16	0.00357	0.00347	0.00153					
85	0.0203	0.0198	0.0087					[13]
**LSIL-HSIL**								**Reference**
**Age**	**No Vaccine**	**Protected HPV 16/18**	**Protected HPV HR**	**Max**	**Min**			
16	0	0.017	0.018	0.000184	0.1485			[13,63,64]
85	0.069	0.068	0.073	0.000184	0.1485			
**Age**	**LSIL-HPV HR**	**LSIL-HPV LR**	**LSIL-> Normal**	**HSIL-HPV**	**HSIL-LSIL**			**Reference**
15	0.082	0.156	0.1605	0.05	0.0692			[13]
85	0.086	0.0816	0.0816	0.05	0.0692			
**HSIL -> CC**								**Reference**
**Age**	**No Vaccine**	**Protected HPV 16/18**	**Protected HPV HR**					
16	0.000714	0.000412	0.000414					
85	0.1	0.0578	0.0579					[13]
**Transition**	**Probability**	**Min**	**Max**					**Reference**
CC1-CC2	0.437	0.400	0.450					
CC2-CC3	0.535	0.500	0.550					[11,13,61]
CC3-CC4	0.683	0.650	0.700					
**Symptoms**	**Probability**	**Min**	**Max**					**Reference**
CC1	0.15	0.12	0.18					
CC2	0.23	0.2	0.25					
CC3	0.6	0.67	0.73					
CC4	0.90	0.87	0.93					[13]
**Death CC**	**Stage I**	**Stage II**	**Stage III**	**Stage IV**				**Reference**
Year 1	0.0377	0.0537	0.2787	0.5806				
Year 2	0.08511	0.16528	0.26535	0.5				[18]
Year 3	0.07406	0.0909	0.10379	1				
Year 4	0.02993	0.0396	0.10613	1				
Year 5	0.0239	0.0396	0.06313	1				
**2. EFFICACY, COVERAGES SENSITIVITY AND DISABILITY WEIGHTS**								
**Parameter**			**BaseCase**	**Min**	**Max**	**alpha**	**beta**	**Reference**
Efficacy Bivalent Vaccine against HPV 16/18			0.9	0.9	1	124	13.8	[21]
Efficacy Bivalent Vaccine against HPV HR			0.302	0.215	0.381	-	-	[65]
Efficacy Quadrivalent Vaccine against HPV6/11			1	0.637	1	-	-	[22]
Efficacy Quadrivalent Vaccine againstHPV16/18			0.9	0.338	0.984	2.9	0.33	[22]
Efficacy Quadrivalent Vaccine against HPV HR			0.25	0.05	0.409	-	-	[66]
EfficacyTreatment LSIL/HSIL			1			-	-	[29,30]
EfficacyTreatment Genital Warts			1			-	-	Experts
Screening Coverage Women 25 or older			0.7	0.4	1	-	-	[7]
VaccineCoverage			0.8	0.5	1			[20]
SensitivityScreening			0.51	0.12	0.99	3.8	.2	[67-69]
SpecificityScreening			0.967	0.91	0.99	25.9	6.0	[67-69]
Treatment proportion			0.864	-	-	-	-	[56]
Disability Weight CC Stage I-II			0.08	0.0	0.2	-	-	[70]
Disability Weight CC Stage III			0.75	0.65	0.85	-	-	[70]
Disability Weight CC Stage IV			0.81	0.70	0.90	-	-	[70]
Disability Weight Death			1	-	-	-	-	[70]
Disability Weight other stages			0	-	-	-	-	[70]
**3. COSTS**								
***MedicalTreatments Costs (USD per year)[34]***					***Care at home costs (USD per year)[36]***			
		Base Case	Min	Max	Base Case	Min	Max	
LSIL		445	334	557	17	13	20	
HSIL		507	380	634	39	31	46	
CCI		1751	1313	2188	309	247	371	
CCII		6867	5150	8583	210	168	252	
CCIII		7143	5357	8928	199	159	239	
CCIV		6698	5024	8373	133	106	159	
Genital Warts		77	58	96	-	-	-	
Screening		22	18	27	-	-	-	
Follow up CC Year 1 and 2		346	259	432	-	-	-	
Follow up CC Year 3 to 5		306	229	382	-	-	-	
Follow up survivors		357	268	446	-	-	-	
Staging		544	408	680	-	-	-	
***Transport Costs (USD per year)[38]***								
		Base Case	Min	Max				
LSIL		32	26	42				
HSIL		35	28	35				
CCI		961	196	5152				
CCII		1368	292	7675				
CCIII		1498	306	8032				
CCIV		3544	108	2847				
Genital Warts		11	6	9				
Screening		2	2	3				
Follow up CC Year 1 to 2		370	76	1986				
Follow up CC Year 3 to 5		204	42	1093				
Follow up survivors		204	42	1093				
Staging		807	36	54				
Vaccination		7	6	8				
***Vaccines Cost (USD per vaccinated Girl)[35]***								
		Base Case	Min	Max				
BivalentVaccineCost		214	133	487				
QuadrivalentVaccineCost		188	157	282				
***TotalCosts(USDperyear***)								
		Base Case	alpha	lambda				
LSIL		780	13829.76	1.2E-01				
HSIL		676	9.09	6.1E-06				
CCI		3717	9.09	7.0E-06				
CCII		9141	9.09	1.3E-06				
CCIII		9602	9.09	5.2E-07				
CCIV		11733	9.09	4.9E-07				
Genital Warts		104	9.09	4.0E-07				
Screening		24	9.09	4.6E-05				
Follow up CC Year 1 to 2		850	9.09	1.9E-04				
Follow up CC Year 3 to 5		587	9.09	5.6E-06				
Follow up survivors		826	9.09	8.1E-06				
Staging		1639	9.09	5.8E-06				
Vaccination Bivalent		258	9.09	2.9E-06				
Vaccination Quadrivalent		232	7.42	1.5E-05				

(Transition probabilities are annual)

#### Transition Probabilities

Probabilities of transition throughout the natural history of the disease were also obtained by means of a systematic search in three pre-selected databases, namely, Pubmed- MEDLINE, Lilacs and Embase. 

A key requirement was to collect transition probabilities specific to the Colombian population. After this search, three transition probabilities were found in studies performed in Colombia [16-18]. Then, for probabilities that had not been assessed in studies conducted in Colombia at the time of the search, data reported in other cost-effectiveness studies published elsewhere in the world would be used appropriately. The methodologies of a previous cost-effectiveness study for CC screening which was carried out in Colombia were found to be the most relevant reference for the rest of the transition probabilities, since that Markov Model had already been calibrated with reference to the Colombian population [13]. In addition, some cost-effectiveness studies from the rest of the world were found to report different values for the same probability. None would be discarded but the minimum and maximum among them would be considered in the sensitivity analysis. 

It is known that prevalence of HPV types is distributed differently among countries. Also, the risk of causing CC is different between HPV types. Therefore, in order to represent the Colombian population as faithfully as possible, and consequently to assess the impact of vaccination in this country, it was considered important to obtain the probabilities for the local population that would discriminate for virus type and evaluate the efficacy profile of each vaccine not only for viral types 16/18, but also for all the other high-risk types.

Given that the difficulty of finding such specific probabilities was foreseen from the beginning, the following strategy was suggested in order to adjust general probabilities found in the literature to the infection profiles of the Colombian population, based on the data supplied by the National Cancer Institute [19]. 

Consider, for example, the probability of progression to LSIL in women infected with HPV 16/18, where this probability may be P_LSIL/(HPV16/18)_. Given that this probability is not available in the literature specifically for the Colombian population, it was derived from the following equation: 

$$P_{LSIL/(HPV16/18)}=\frac{R_{2}}{R_{1}}\times P_{LSIL/HPV}$$

Where:

• P_(LSIL/HPV)_= Probability of progression to LSIL for women with infection caused by HPV of any type
• R_1_= Proportion of women infected with HPV 16/18 among the total number of infected women
• R_2_= Proportion of LSIL cases attributed to HPV 16/18

The probability of death from any cause was derived from the National Department of Statistics (DANE for its acronym in Spanish) mortality data for different ages above age 12 for the year 2008. Additionally, the coverage of Pap cytology testing in the domestic context was derived from the National Health and Demographics Survey conducted by Profamilia in 2010 and vaccination coverage was obtained from national records regarding immunization of children in 2010 [20].

#### Efficacy of strategies and treatments

The efficacies of the interventions were also obtained through systematic literature searches. They included sensitivity and specificity of the Pap test, as well as efficacy and safety of each of the vaccines in the study. A search was also conducted for the efficacy in the treatment of pre-neoplastic cervical lesions. Confidence intervals of efficacy measures retrieved by systematic searches were used to conduct sensitivity analyses. 

As far as efficacy values for vaccines are concerned, the papers selected were those that fulfilled the inclusion criteria and whose study participants reasonably matched the hypothetical population of this model. There are neither meta-analyses nor head-to-head clinical trials comparing the two vaccines. The best approximation to the target population in this study (12 years old girls without sexual debut) and the end point needed (persistent infection of up to 6 months with HPV16/18) was found in two clinical trials, one per vaccine type [21,22]. As no study has demonstrated differences in the relative efficacy of bivalent compared with quadrivalent vaccine, a baseline analysis assumed the same efficacy of 90% for both agents, as used elsewhere [23-25], and probabilistic sensitivity analyses were carried out considering confidence intervals of the corresponding efficacy measures. Cross-protection of both vaccines and efficacy against HPV 6/11 for quadrivalent vaccines were obtained by systematic search and are reported in Table 1.

Moreover, it has been determined that the safety profile of the two vaccines is very favorable [21,22,26-28], so the inclusion of adverse events in the model was not considered necessary.

By the same token, since the efficacy of the treatments for pre-neoplasic lesions has been found to be close to 100% [29,30] for simplicity it was decided to use the assumption that all women treated for cervical intraepithelial neoplasia would be cured.

Cytology sensitivity and specificity was considered in the model. In this way, the cost of treating false positive cases was accounted for in each state of the model. Also, for false negative cases, the impact of lack of treatment in both costs and health results was included. 

#### Cost Measurement

This economic study was framed within a societal perspective, as recommended by most of the guidelines for economic studies, including the WHO guidelines [15,31].

In Colombia, the Methodological Guidelines for the Development of Comprehensive Care [32] recognize the advantages of the social perspective and propose an alternative limited societal perspective that includes the expenditure of the third party payer in addition to the out-of-pocket spending by the patient as a result of the intervention. Consequently, in following these guidelines, the costs listed below were considered:

- **Medical costs paid by the health sector**:
  • Cost of the procedures: Medical expenses to treat any disease state of the model. It includes health care personnel fees; treatments such as surgeries, radiation, chemotherapy, etc.; hospitalizations; diagnostic tests, and others.
  • Cost of the medication.
  • Disability costs: Compensation for disability days, charged to the insurance company.
- **Costs paid by the patient and the family**: transportation costs, home care costs, cost of the patient and accompanying person’s time, cost of sliding-scale fees.

In principle, the cost methodology consisted of developing ‘Typical Cases’ for each ‘stage of intervention’ during the natural progression of the disease. These were developed on the basis of clinical practice guidelines in place in the country [14,33]. It is worth noting that in absence of management guidelines for genital warts, the typical case was constructed by consulting gynecologists. 

Most of the selected sources of costs were government databases, national fees manuals, and national DANE studies and surveys [34-39].

Whenever they were available, minimum and maximum values reported in the literature sources were used for the sensitivity analyses. Otherwise, ±20% intervals were used for this purpose. 

On the other hand, applying the criterion suggested in the Methodological Guidelines for the Development of Comprehensive Care [32] to avoid considering non-consequential costs, it was decided not to include other costs such as lodging and food for the accompanying person while the patient is hospitalized. There is not enough information to determine these costs with certainty and their impact is not expected to be substantial.

The costs of the vaccination program, including transportation, storage or product losses were included in the model during the sensitivity analysis.

Additionally, and as recommended by the experts [32,40], productivity losses were included in the denominator as a measure of effectiveness measured in time, not as an additional cost.

A 3% discount rate was applied together with a 0-6% interval for the sensitivity analysis. It was applied to costs and to health outcomes. 

### Model Calibration

To ensure that model was actually representing the natural history of disease in Colombia a calibration procedure was performed. This calibration was expected to minimize any possible bias introduced when selecting probabilities used in models for other countries and when modifying those probabilities to be adjusted to the Colombian population, as discussed above. 

**Figure 2:**
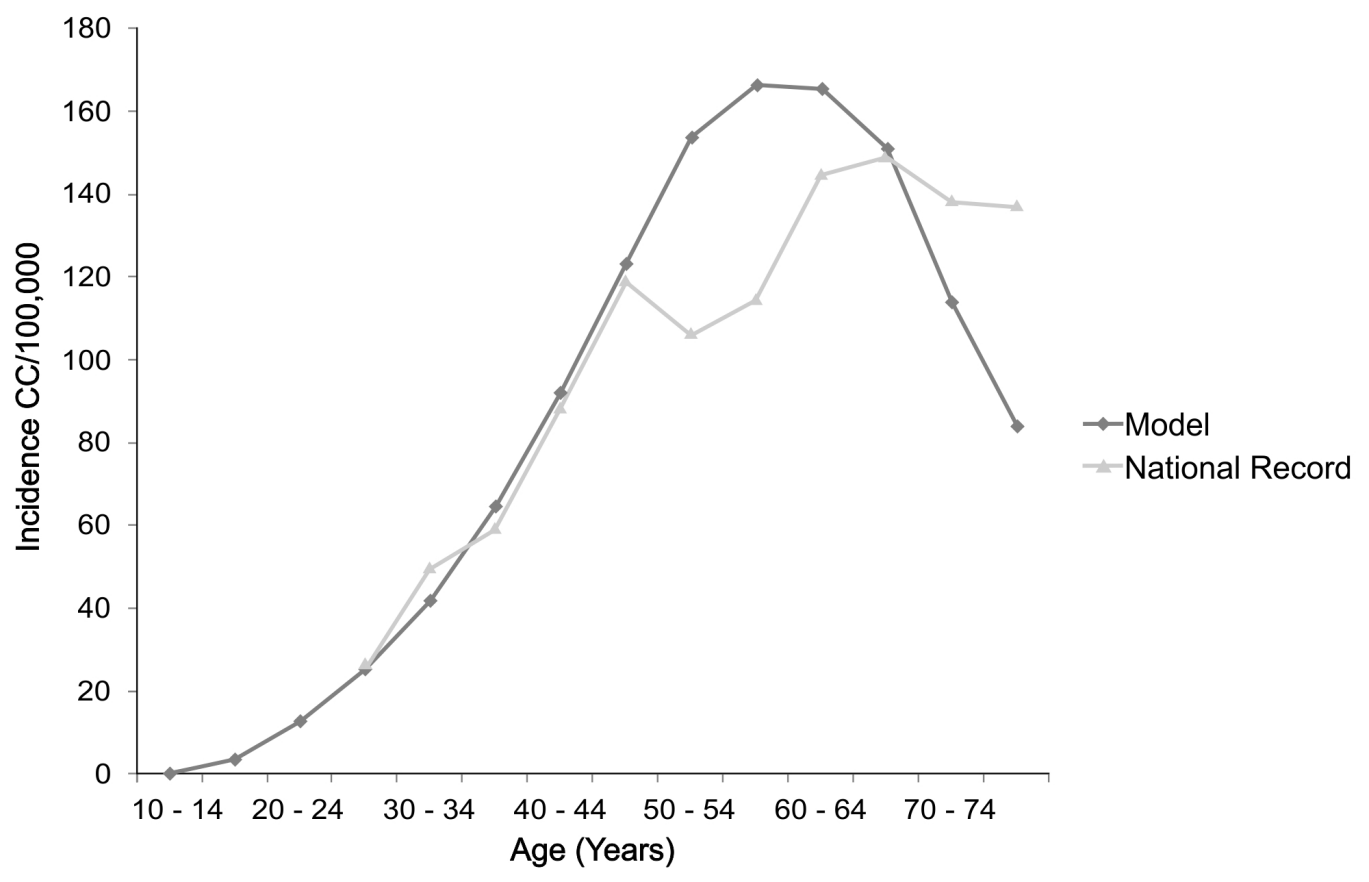
Comparison of the incidence calculated in the Model and the incidence reported in the National Register. Both curves represent incidence by five-year groups. Source: Author.

For calibration, the model was run without any intervention, and the number of the resulting cancer cases, by age, was compared with the Cancer Population Registry of Cali, Colombia, between 1962 and 1966 [41]. Since in the preliminary evaluation of the model no effect of any of the interventions analyzed in the study (vaccination or screening) would be considered, a registry of incidence dating back before 1990 was used, considering that this was the first year of the *National Program for the Control and Early Detection of Cervical Cancer* [8]. This comparison is showed in Figure 2. 

After comparing the results obtained with the official registry, options for modifying the model or the parameters were then considered. The model with the parameters derived from the systematic search was preserved to the greatest extent possible so as not to introduce arbitrary modifications that would undermine its veracity. An effort was made to keep modifications to a minimum. 

A Chi-square goodness of fit test with 14 degrees of freedom on the Observed versus Expected Model frequencies was done and the difference was not significant (P<0.20). Consequently, it was deemed appropriate to use the health state transition probabilities found in the literature (see Table 1). 

### Cost-Effectiveness Analysis

Two cost-effectiveness measurements were calculated:

- Average cost-effectiveness ratio (ACER)[40,42,43]:

$$ACER_{a}=\frac{C_{a}}{E_{a}}$$

Where:

C_a_= Costs associated with intervention *a*, measured in US Dollars

E_a_= Effectiveness value associated with intervention *a*, measured in DALYS

Incremental cost-effectiveness ratio (ICER)[40,42,43]

$$ICER_{ab}=\frac{C_{a}-C_{b}}{E_{b}-E_{a}}$$;

given that *a* is the new strategy to be tested and *b* is the current practice.

Where

C_x_= Costs associated with intervention *x*, measured in US Dollars

E_x_= Effectiveness value associated with intervention *x*, measured in DALYS;

Averted DALYS = *b* strategy DALYS- *a* strategy DALYS; given that *a* is the new strategy to be tested and *b* is the current practice.

An intervention strategy is considered dominated if it was found to be more costly and less effective or less effective and less cost cost-effective than another.

The ICER was analyzed using the World Health Organization CHOICE methodology, according to which, every time an ICER is lower than the per capita Gross Domestic Product (GDP), the strategy is considered ‘*Very cost-effective*’ or rather, as having a very convenient cost-effectiveness profile. On the other hand, if the ICER is between one and three times the per capita GDP, the strategy is considered ‘*cost-effective*’, i.e., as having a good cost-effectiveness profile. Finally, if the ICER is higher than three times the per capita GDP, the strategy is considered as having a poor cost-effectiveness profile, and is designated ‘non cost-effective’ [44]*.*

For this analysis, thresholds (1*per capita GDP and 3*per capita GDP) were determined using World Bank data reported for Colombia in 2010[45,46], resulting US$ 6,224 and US$ 18,673, respectively.

### Sensitivity Analysis

As explained above, uncertainty was considered in some of the parameters included in the model, such as transition probabilities, costs and efficacy of strategies. Besides, one-way sensitivity analyses were conducted to assess impact of such uncertainty in final results. In addition, probabilistic sensitivity analysis with 1000 iterations was conducted for the following parameters: (i) efficacy of vaccination; (ii) Sensibility/ Specificity of Pap Test; (iii) Costs and (iv) Discount for both costs and health results. For all parameters the Beta distribution was assumed, except costs, whose distribution was assumed to be Gama. The distribution parameters are in Table 1.

### Other Analyses

Moreover, other scenarios of interest for the evaluation were also considered, as follows:

- Coverage of the vaccination program: between 50% and 100%.
- Interruption of the screening program: this possibility was evaluated, with a variation down to 0% in cytology coverage.
- Duration of immunity: hypothetical scenarios were considered where proposed immunity lasts only 10 and 30 years after vaccination.
- Cross protection: cross protection reported to this date in clinical trials was considered. For this purpose, the efficacy of the vaccine for HPV 31/33/45/52/58 and its effect on the results were included.

## Results

### Result of the Baseline Cost-Effectiveness Analysis

**Figure 3:**
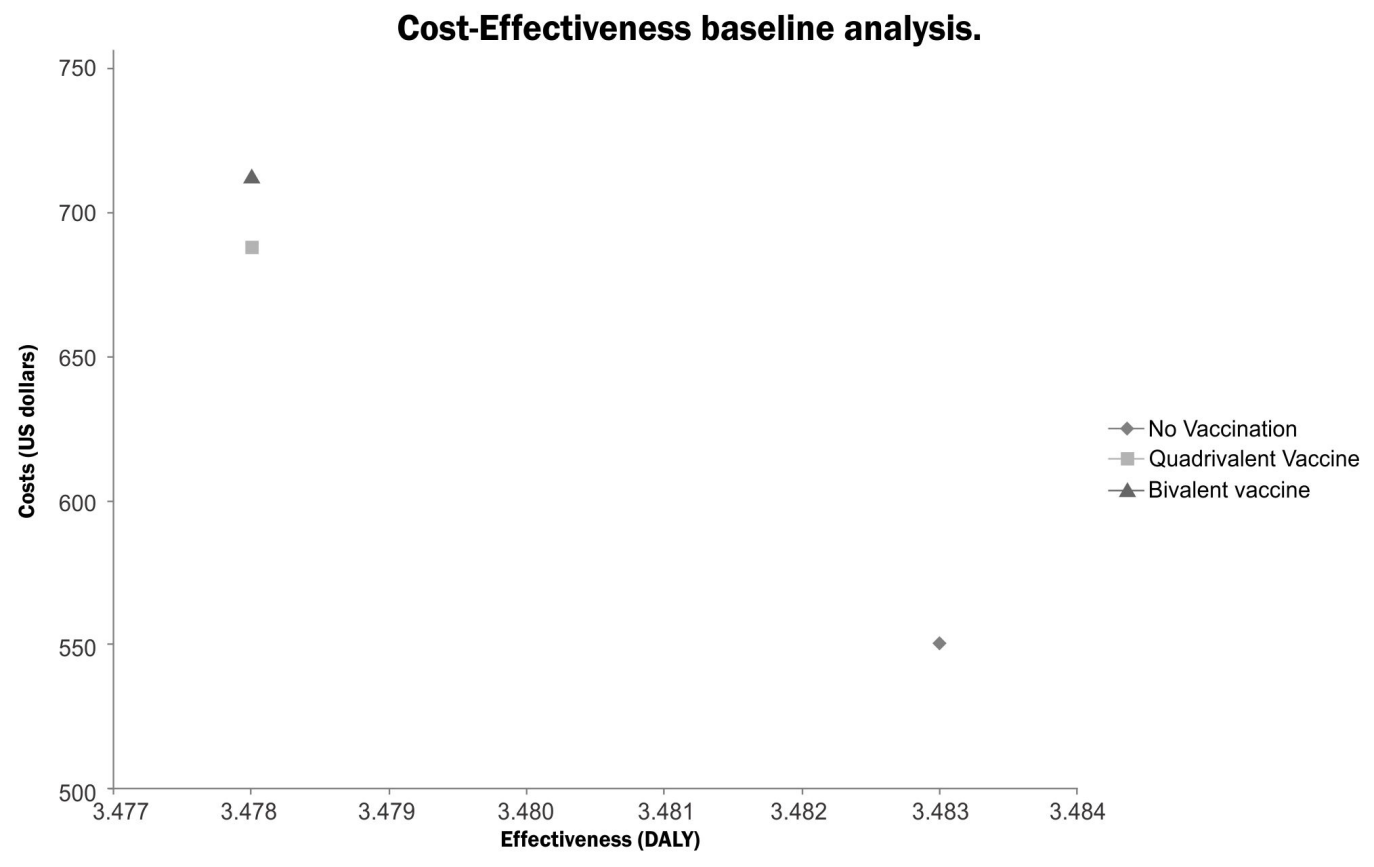
Cost-Effectiveness baseline analysis. Source: Author. This represents the relationship between cost and effectiveness for assessed alternatives.

The baseline analysis using the main parameters revealed that the most costly strategy is the bivalent vaccine, followed by the quadrivalent vaccine, and lastly, screening with no vaccination. The same order was found for the effectiveness results, where the bivalent and quadrivalent vaccines were estimated to be more effective than screening without vaccination. The results are shown in Table 2 and Figure 3. 

**Table 2:** Results of the baseline cost-effectiveness analysis.

**Name of the Strategy**	**Costs (US dollars)**	**Effectiveness (DALYS)**	**Average C-E Ratio¥**	**Incremental Costs**	**Incremental Effectiveness(Averted DALYS**)	**Incremental C-E Ratio ∞**
No Vaccination	551	3.483	158			
Quadrivalent Vaccine	688	3.478	198	137	0.00563	24241
Bivalent vaccine	713	3.478	205	162	0.00563	28765

¥ Strategy costs divided by DALYS

∞ Strategy Costs - ‘No vaccination’ Costs / ‘No vaccination’ DALYS - Strategy DALYS

In accordance with the WHO CHOICE methodology, in the base case analysis it was determined that HPV vaccination, either with the bivalent or the quadrivalent vaccine, is a ‘non cost-effective’ strategy. The ICERs were estimated to be 24,241 USD/Averted-DALY for the Quadrivalent Vaccine and 28,765 USD/Averted-DALY for the Bivalent vaccine relative to screening; both values are higher than 3 times GDP per capita for Colombia (18,673 USD) which is often recommended as an appropriate maximum willingness to pay threshold value.

### Sensitivity Analysis

Considering one-way sensitivity analyses, it was also noted that cost variations within the defined values did not result in significant modifications of the cost-effectiveness profiles of the alternatives evaluated. However, when variation in the cost of the vaccines was considered, it was noted that there was a considerable impact on the cost-effectiveness profile. Table 3 shows how at extreme price values, one vaccine can be dominated by the other one. Within reasonable sensitivity analysis intervals, neither vaccine is consistently more cost-effective.

**Table 3:** One way sensitivity analysis and other analysis results.

**Comparison**	**Parameter**	**Value**	**ICER**	**Comparison**	**Parameter**	**Value**	**ICER**
QV Vs. NV	Efficacy Bivalent Vaccine	0.9	24210	BV Vs. NV	Cost Bivalent Vaccine	one hundred and thirty-three US Dollars	17258
BV Vs. NV			28727	QV Vs. NV			24210
BV Vs QV				BV Vs QV			QV dominated
QV Vs. NV		1	24210	QV Vs. NV		four hundred and eighty-seven US Dollars	24210
BV Vs. NV			25048	BV Vs. NV			67404
BV Vs QV			32548	BV Vs QV			BV dominated
BV Vs. NV	Efficacy Quadrivalent Vaccine	0.338	28727	QV Vs. NV	Cost Quadrivalent Vaccine	one hundred and fifty-seven US Dollars	19763
QV Vs. NV			80519	BV Vs. NV			28727
QV Vs BV			847	BV Vs QV			BV dominated
QV Vs. NV		0.984	21439	BV Vs. NV		two hundred and eighty-two US Dollars	28727
BV Vs. NV			28727	QV Vs. NV			37549
BV Vs QV			56118	BV Vs QV			QV dominated
QV Vs. NV	Discount Of Costs	0	9262	QV Vs. NV	Discount of Health Outcomes	0	6162
BV Vs. NV			14338	BV Vs. NV			7311
NV Vs. BV		6%	29042	BV Vs. NV		6%	80795
NV Vs. QV			33308	QV Vs. NV			95871
QV Vs. NV	Vaccination Coverage	0.5	24300	QV Vs. NV	Protection Duration	30 Years	40082
BV Vs. NV			29069	BV Vs. NV			46757
BV Vs. NV		1	24245	QV Vs. NV		70 Years	24210
QV Vs. NV			28629	BV Vs. NV			28727
QV Vs. NV	Screening Coverage	0	4976	QV Vs. NV	Cross Protection		13835
BV Vs. NV			5942	BV Vs. NV			16986
QV Vs. NV		1	32462	NV: Non Vaccination – Screening Alone; BV: Bivalent Vaccine; QV Quadrivalent Vaccine; A vs. B means that B is used as reference to calculate the ICER.			
BV Vs. NV			38761				

 It was found that in order for the quadrivalent vaccine to become a ‘cost-effective’ alternative in comparison with screening, it must be priced at US$ 49 or less per dose. A price of US$ 19 per dose would be necessary for this alternative to become ‘Very cost-effective’.

Regarding the bivalent vaccine, to be ‘cost-effective’ when compared with screening, the maximum price per dose of bivalent vaccine should be US$  47, and to be ‘Very cost-effective’ a price of US$ 17 would be necessary. 

Considering that the quadrivalent vaccine has additional protection against genital warts, if its price is the same reported in 2010 (USD188/ vaccinated girl), the bivalent vaccine should cost US$ 183 per vaccinated girl to be as cost-effective as the quadrivalent vaccine.

In terms of transition probabilities, it was noted that reasonable changes in parameter values do not have a high impact on the results. However, it was found that results may change when the risk of developing LSIL and moving on to develop HSIL is modified. In general, to the extent that risks are increased, vaccines become more cost-effective, with an ICER slightly below the 3 times per capita GDP threshold or higher with relatively small increases in disease progression risks (Results available from authors on request).

On the other hand, if the sensitivity of the cytology test is very low, vaccination becomes a ‘cost-effective’ alternative. By the same token, if the sensitivity of the cytology test increases, vaccines go further into the ‘non cost-effective’ range when compared with screening alone (Data available on request).

It was also observed that the quadrivalent vaccine dominates the bivalent vaccine when its effectiveness reaches the maximum value (0.984), although it does not become a ‘very cost-effective’ alternative. When the effectiveness of this vaccine drops below 0.5, it conversely becomes dominated by the bivalent vaccine. 

Regarding the time discounting of costs it was found that the vaccination with both vaccines became ‘cost-effective’ when rate was 0%. It was also noted that when the discount rate of benefits is between 0 and 1%, averted DALYS increased to such an extent that the bivalent vaccine becomes a ‘very cost- effective’ alternative, just like the quadrivalent vaccine.

The results derived from one-way sensitivity analysis are shown in Table 3.

**Figure 4:**
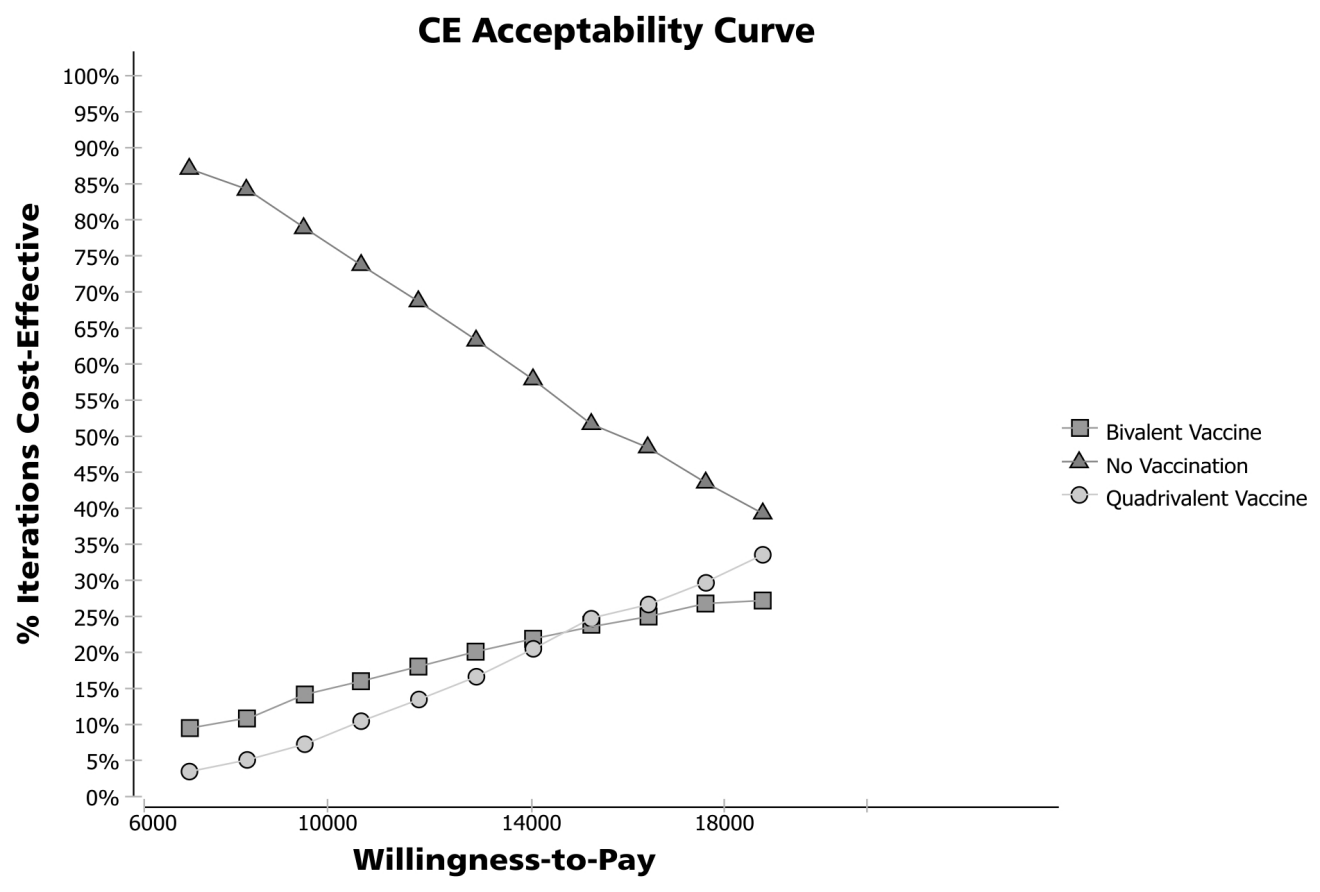
Probabilistic Sensitivity Analysis: Acceptability Curve. Source: Author.

With probabilistic sensitivity analysis it was found that ‘No-vaccination’ can be the chosen alternative in 39.4% of iterations, with a threshold of 3 GDP per capita. Also, quadrivalent and bivalent vaccines resulted in ICER values below 3 GDP per capita in 33.4 and 27.2% of iterations, respectively. However, with a threshold of 1 GDP per capita, ‘No vaccination’ is the selected alternative in 87.2% of iterations. Acceptability curves are shown in Figure 4.

### Additional Analyses

Variations in the coverage of the vaccination program create a low-level impact on the results. On the other hand, when coverage of the screening program reaches a level of 0-20%, the result is that the bivalent and quadrivalent vaccines become ‘cost-effective’ alternatives. At higher levels of screening coverage vaccination becomes a ‘non cost-effective’ alternative.

Baseline analysis assumed that both vaccines provide immunity for an entire lifetime. If vaccines were deemed ‘non cost-effective’ in such analysis, any reduction in lifetime immunity requiring booster vaccination would make the vaccines’ cost-effectiveness even more unfavorable.

Finally, when cross protection is conferred to the two vaccines in the model, there is a reduction in the ICER (as compared with screening) that makes both alternatives cost- effective.

These results are also shown in Table 3.

## Discussion

As a form of health intervention, vaccination has been shown to reduce medical costs substantially in different countries. *Global Alliance for Vaccines and Immunization* has determined that most vaccination campaigns cost less than US$ 50 per year of life saved, whereas providing treatment for diseases such as hypertension may create costs to a level ranging between US$ 4,000 and US$ 90,000 [47].

As far as vaccination in Colombia is concerned, other strategies classified as ‘very cost-effective’, with an ICER lower than the per capita GDP, have been found recently. Such is the case of the conjugated pneumococcal vaccine [48],the tetanus vaccine[49] and the Pneumovax 23 in adults over 28 years of age [50].

This study compared screening with two HPV vaccines available in the market, using a Markov model with probabilities adjusted to the local population. This way, the long term progression of this disease was represented in the model, which made it possible to assess the impact of vaccination, even though a decrease in disease incidence is expected to be observed 15 years after vaccination or later. 

To our knowledge, this is the first published cost-effectiveness study with such a close approximation to Colombian specific population. The effect of introducing the HPV vaccination on mitigating the burden of the disease had already been evaluated, though not from the perspective of a cost-effectiveness study [51]. Also, although in 2008 Goldie et al. published a cost-effectiveness study of HPV vaccination which covered six countries including Colombia, their model may not represent the specific Colombian reality, considering that probabilities included data for the entire Latin American population and the screening programs proposed did not match the Colombian situation. Additionally, costs were derived by approximation, from studies conducted in other countries, using GDP and other indicators [52]. Consequently, ours is the first cost-effectiveness study that comes closer to recreating the current characteristics of the Colombian population.

In contrast with studies conducted previously for other vaccines in Colombia, the baseline analysis led to classifying HPV vaccines as ‘non cost-effective’ when compared with the existing screening approach. Moreover, the quadrivalent vaccine was more cost-effective than the bivalent vaccine, especially because of the money saved by reducing the number of genital warts cases to be treated. 

This model turned out to be very sensitive to the cost and efficacy of the vaccines. Vaccines never dominated or were dominated by screening within the ranges studied. However, the ICER positions of the vaccines did change, when the parameters for cost and effectiveness were modified. In terms of cost, lower prices per dose of the quadrivalent vaccine would make it dominant over the bivalent vaccine, as is also true when its efficacy comes close to the maximum value reported in the literature. This points to the need for paying close attention to the dominance relationships between the two vaccines.

Although the cost of the vaccines was taken from official databases, considered the best sources available on the grounds of impartiality and ability to represent the range of medication costs, prices will always be subject to trade conditions and to agreements between suppliers and buyers. Differential negotiations with the suppliers of these vaccines may change their cost-effectiveness profile. Although they will probably continue to be more costly and more effective than screening, the price relationship between the two vaccines will vary.

As far as the impact of efficacy is concerned, the study shows an overlap between the two vaccines in terms of the confidence intervals for this measurement. In view of this, there is not sufficient evidence to assert that any vaccine is more effective than the other one. Given this uncertainty, the most appropriate thing would be to conduct a randomized clinical trial comparing the two vaccines in a similar population of 12 year-old girls, in order to measure efficacy in terms of the number of HPV infections prevented. This way, the two vaccines would be compared head-to-head in the same scenario, thus reducing uncertainty in the analysis.

There are previous cost-effectiveness studies in the scientific literature comparing the two vaccines. In Ireland, a study used the assumption of 90% efficacy for the two vaccines, and the price of the two vaccines is the same in that country. Although the two vaccines were shown to be ‘very cost-effective’ alternatives according to the threshold already established by the Irish government, the quadrivalent vaccine showed the best cost-effectiveness profile. In order to have the same ICER for the quadrivalent vaccine, the bivalent vaccine had to be 22% less costly [25]. Similar results were obtained by Brisson et-al in a cost-effectiveness analysis for Canada. By assuming efficacy of 95% and cost of Canadian Dollars (CAND) 400 for both vaccines, once again the quadrivalent vaccine was deemed more ‘cost-effective’ than the bivalent (ICER = CAND21000/QALY and CAND31000/QALY respectively) [53]. Also, Jit et al found that in order to be cost-effective the bivalent vaccine should be cheaper considering its lack of protection against genital warts [24]. In all these studies, genital warts were accounted for not only in terms of treatment savings but also in terms of QALYS, so the result was to be expected considering the advantage of the quadrivalent vaccine. For the local Colombian context, a result of this type is completely plausible, even more when considering the uncertainty already described. 

In view of the above, this analysis enabled us to clearly identify the relationship between screening and vaccination in terms of cost as well as effectiveness. However, in the analysis between vaccines, some sources of uncertainty were identified and, consequently, the results should be interpreted with caution. The model was developed using the best scientific evidence available, but there are weaknesses in knowledge that preclude more reliable conclusions. 

To become a ‘cost-effective’ alternative for Colombia we found that a vaccine price reduction would be necessary. The prices considered in this study (US$ 188 for quadrivalent and US$ 214 for bivalent per vaccinated girl) should be reduced about 32-36% to consider these vaccines as cost-effective alternatives in the local context. 

The results found here are consistent with cost-effectiveness analysis of HPV vaccination performed for other countries, even when vaccines have been found cost-effective in other scenarios. In a Canadian study with similar assumptions to this one, Brisson et-al reported an ICER of US$  21,000- 31,000/QALY [53]]. For UK, Jit et-al calculated an ICER of USD44,321/QALY[23], and in a review of cost-effectiveness analysis for USA, it was found that ICERs can reach USD24,300/QALY[54]. Even though in all these cases vaccines were classified as ‘likely to be cost-effective alternatives’, this result is opposite to the classification obtained in this study because of the difference in considered societal willingness-to-pay thresholds. For such developed countries, thresholds can reach values of US$  150,000 whereas for Colombia three times GDP per capita is about US$ 18,700. This difference, added to other ones in the design of the other studies such as including herd protection and measuring health results using QALYs, can explain why these results differ from studies previously published.

In addition, WHO’s proposed price of US$ 10-25 per vaccinated girl supports the idea of reducing the price [5], along with an analysis of Goldie et-al about HPV vaccination in Latin America, which suggests that for these countries, the prices of vaccination currently set in Europe and North America are not appropriate [52]. These prices are near the ones considered in the base case analysis of this study. A reduced price would be also consistent with prices of other vaccines in Colombia, like pneumococcal and tetanus vaccines, which are deemed ‘very cost-effective’ at prices per dose between USD1.4 and US$ 14 [49,50].

As in previous studies, it was found that when screening coverage is reduced, the impact of vaccination is greater [55]. In Colombia, it is reported that for 2010 61% of women between 18 and 25 years went for a Pap cytology every year, and 6% every three years. However, for Caribbean region within the country, coverage can drop to 47%. In Guajira department, for example, only 37% of women go yearly for Pap Cytology [7]. By the same token, the cost-effectiveness profile of the vaccines is improved when the sensitivity of the Pap cytology testing is reduced. Therefore, this may suggest greater impact of vaccination in regions of the country where the health systems are not well developed and, consequently, the performance and coverage of the cytology tests is low. 

Additionally, it is important to mention that the model included the percentage of diagnosed women who underwent treatment, obtained from previous studies [56]. According to this, 86.4% of diagnosed women were treated in the model, which adjusted the analysis to Colombian situation in a closer way and permits the consideration of health system characteristics that could impact HPV vaccination efficacy.

One weakness of this work is the fact that it is a predictive theoretical model based on data reported in the scientific literature and not on specific clinical findings pertaining to CC, the natural history of the disease, or the associated prevention and promotion programs. A pilot trial of vaccination would allow considering other variables that are outside the scope of this work. 

Additionally, it would be important to collect cost data not on the basis of a typical case but, better still, from a real-world study designed to follow these patients and to determine how much they and their families spend for the treatment of the disease. 

Finally, three additional analyses which were not performed for this study should be discussed. The first one is male vaccination. Previous analyses have found that vaccinating boys in addition to men is unlikely to be ‘cost-effective’ [23,57], and considering also that this has not yet been approved for Colombia, such an analysis is not currently considered of major relevance for this health technology. 

Secondly, the effect of HPV vaccination in Human Immunodeficiency Virus (HIV) or Acquired Immunodeficiency Syndrome (AIDS) population was not included here. Even though epidemic in Colombia is considered as concentrated and low [58], this analysis was not possible because of lack of information By the moment of this analysis, no assessment of safety or efficacy in immunocompromised population had been published. An initial consideration of different variables suggest that vaccination would be less effective in HIV/AIDS population, considering that distribution of HPV types is different in HIV-infected population. Since HPV16 is present in lower rates than in the rest of the population, and other types incidence is increased, vaccine efficacy can be reduced [59]. Also, it has been found that HPV related lesions are 5 times less likely to regress in HIV- positive women than in those without HIV [60]. However, the available information about efficacy in this subgroup of population was deemed as ‘not-enough’ to propose further assumptions in the model that could result in reliable conclusions. For this reason it is suggested that in further assessments of HPV vaccination for Colombia this variable could be included, if information is available. 

On the other hand, herd protection is suggested to be included in further analysis, since this consideration can influence the final results. For example, Elbasha et-al described that if herd protection is not taken into account, along with protection against genital warts, the ICER can increase from USD 3,000/QALY to USD 214,000/QALY [9]. So, by considering herd protection in the local context, vaccination strategies could be potentially favored and different results could be expected. Given this, assumptions about HPV infection transmission should be closely evaluated to avoid results that are not representative of the actual epidemiologic and demographic conditions, leading to over- or underestimation of the real vaccination costs and effects.

## Conclusions

Since 2009, the WHO has stated its position regarding HPV vaccination. It recommends including vaccination whenever the prevention of CC is a public health priority, as long as programs are viable, funding is guaranteed, and the cost-effectiveness profile is taken into consideration in the country, or at least in the region [5]. This work is an attempt to come closer to fulfill the latter premise. The cost-effectiveness profile of the two vaccines is now available from a societal perspective, based on the best information available to date for recreating the local scenario as carefully as possible. Even though it was found that vaccination may be classified as ‘non cost-effective’, threshold vaccine prices were calculated that would allow these vaccination programs to become cost effective. Consequently, any future decisions will depend on price considerations as well as on the analysis of the program and on the budgetary impacts. 
